# Supplementary material for: Caffeine blocks SREBP2-induced hepatic PCSK9 expression to enhance LDLR-mediated cholesterol clearance
Source: Nat Commun. 2022 Feb 9;13:770. doi: 10.1038/s41467-022-28240-9 (PMC8828868; doi:10.1038/s41467-022-28240-9)
Supplement: Supplementary file 1 — Supplementary Information [file 41467_2022_28240_MOESM1_ESM.pdf]

Supplementary Information

Caffeine Blocks SREBP2-Induced Hepatic PCSK9 Expression to Enhance LDLR-Mediated Cholesterol Clearance

Lebeau et al.

Fig. S1

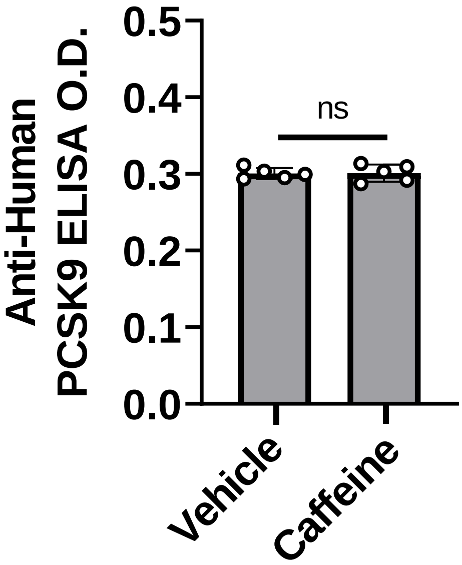

Fig. S1. Caffeine (CF) does not interfere with the anti-human PCSK9 ELISA. Recombinant PCSK9 levels were measured in the presence or absence of CF (200 μM) added directly to the ELISA assay. (n=5 independent samples per group; data presented are mean ± s.d. Statistical comparisons between two groups were conducted using unpaired Student's *t*-tests (ns, non-significant). Source data are provided as a Source Data file.

Fig. S2.

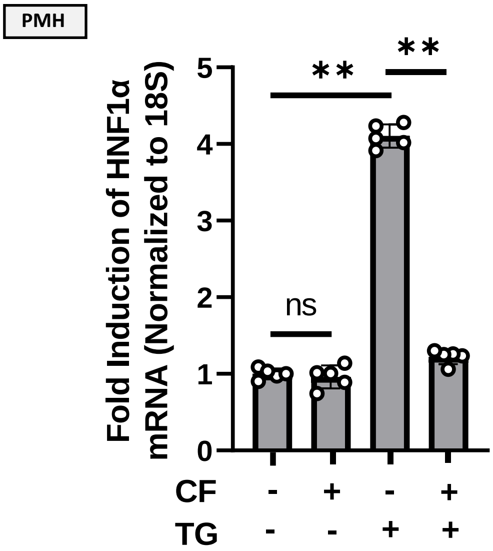

Fig. S2. Caffeine (CF) reduces HNF1α expression in hepatocytes. (A) HuH7 cells were treated with an ER stress-inducing agent, thapsigargin (TG; 100 nM) in the presence or absence of CF (200 μM) for 24 hours. HNF1α expression was measured via qPCR. (n=5 biologically independent samples per group; data presented are mean ± s.d. Statistical comparisons between multiple groups were compared using one-way ANOVAs with the Tukey HSD post-hoc test (*, p<0.05; **, p<0.01). Source data are provided as a Source Data file.

Fig. S3.

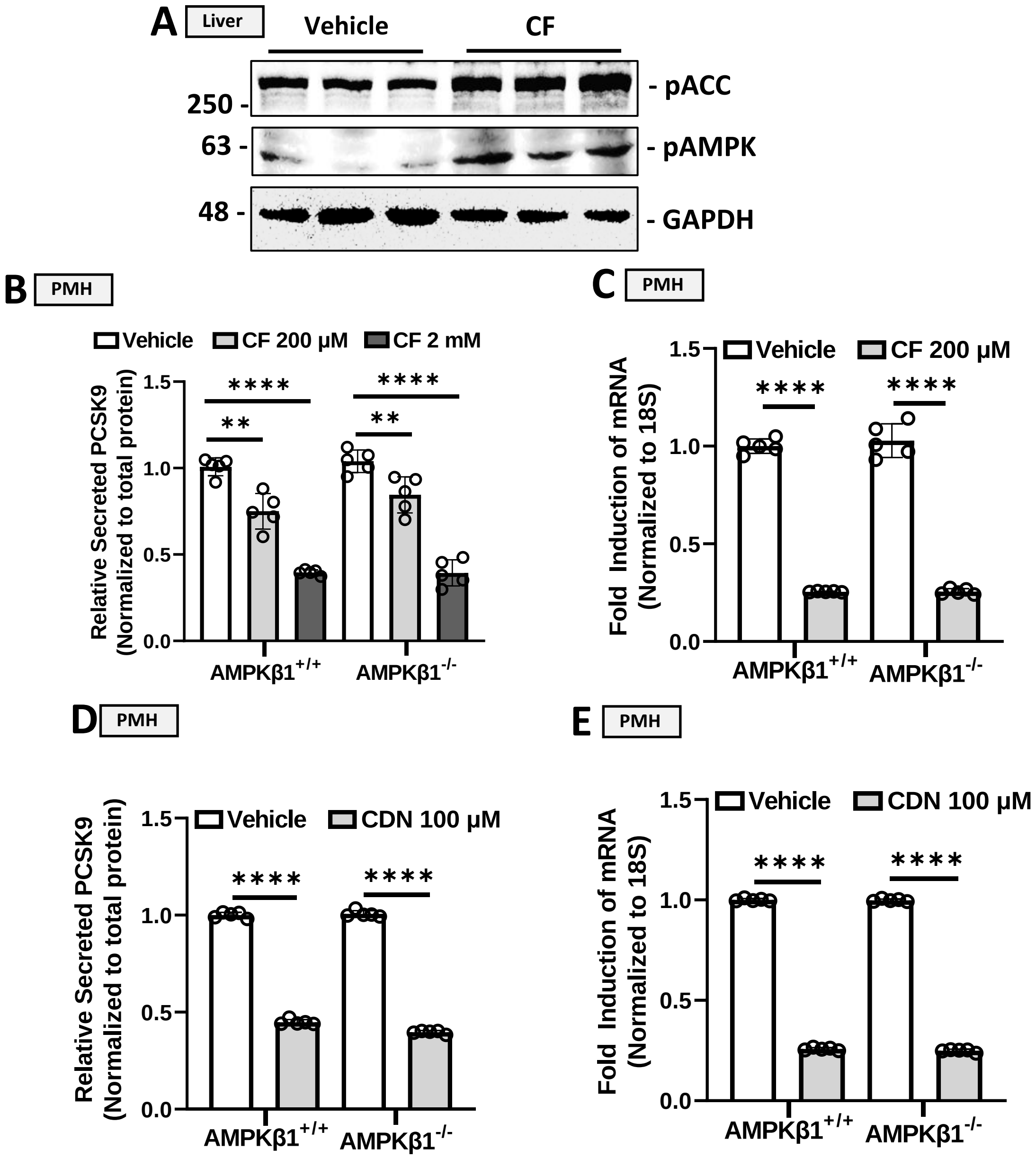

Fig. S3. Caffeine (CF) upregulates AMPK activation, but reduces PCSK9 independent of AMPK activity. (A) 12-week-old male C57BL/6J mice were fasted and treated with CF (50 mg/kg) for 8 hours prior to sacrifice (n=6). (A) The effects of CF on p-AMPK and p-ACC expression were measured via immunoblots. (B) Secreted PCSK9 levels were measured in primary mouse hepatocytes (PMH) in AMPKβ1$^{-/-}$ treated with CF and compared to controls using an ELISA. (C) Relative mRNA levels of PCSK9 were measured to confirm the observations made from the ELISAs. (D) Secreted PCSK9 levels were measured in PMH in AMPKβ1$^{-/-}$ treated with CDN (100 μM) and compared to controls using a ELISAs. (E) Relative mRNA levels of PCSK9 were measured to confirm the observations made from the ELISA. Panels B-E: n=5 biologically independent samples per group; data presented are mean ± s.d. Statistical comparisons between two groups were conducted using unpaired Student's *t*-tests (*, p<0.05; **, p<0.01; ***, p<0.001; ****, p<0.0001). Source data are provided as a Source Data file.

Fig. S4.

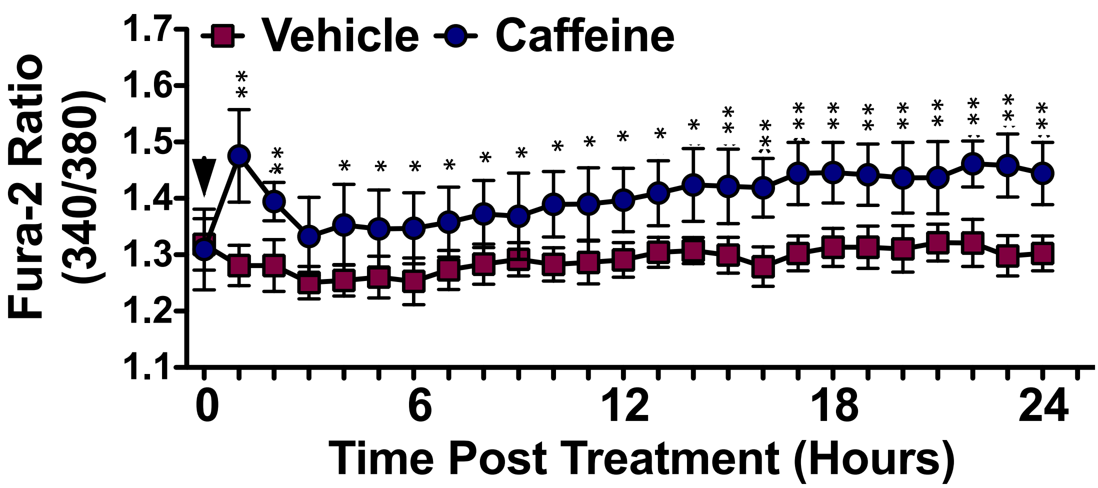

Fig. S4. Caffeine (CF) modulates endoplasmic reticulum (ER) Ca^{2+} levels. HuH7 cells were pre-treated with either CF (200 µM) or vehicle for 24 hours and loaded with the high-affinity Ca^{2+} dye, Fura-2-AM. Exposure of cells to a high dose of TG (1 mM) induced a spontaneous depletion of ER Ca^{2+} (n=3 biologically independent samples per group). Data presented are mean ± s.d.; Statistical comparisons between two groups were conducted using unpaired Student's *t*-tests (*, $p<0.05$; **, $p<0.01$). Source data are provided as a Source Data file.

Fig. S5.

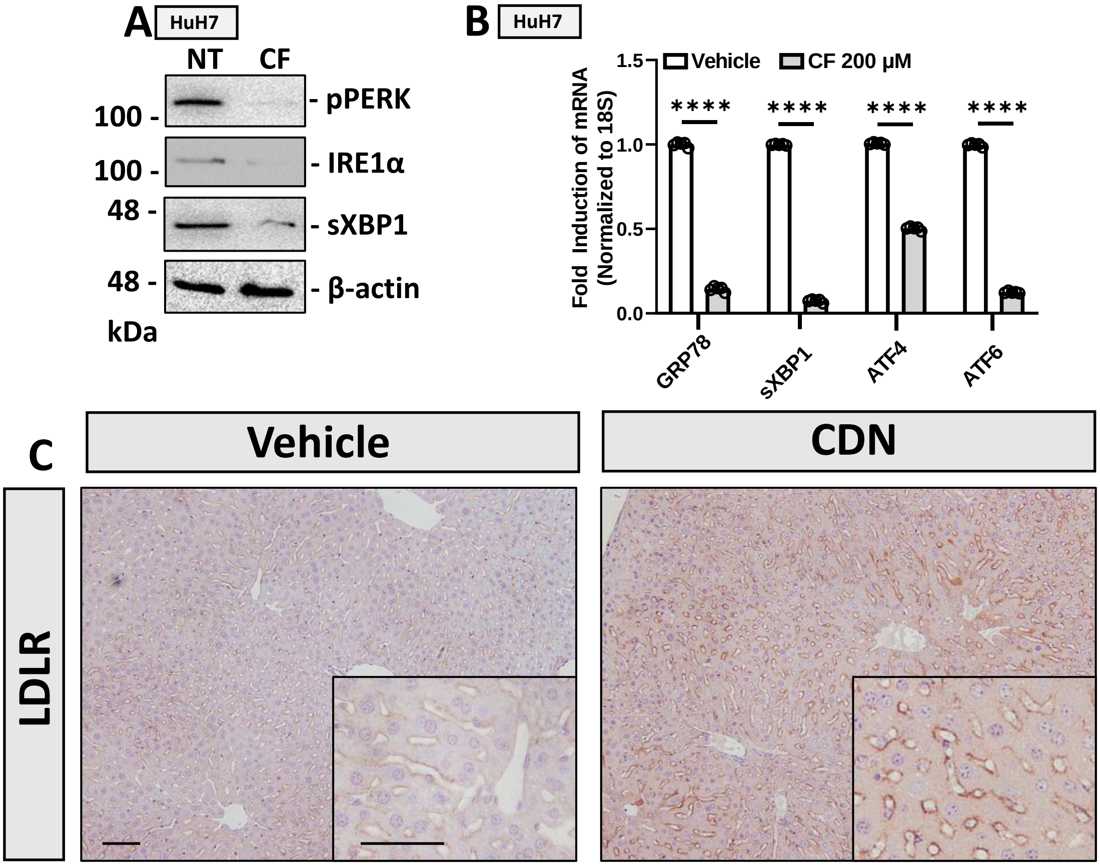

Fig. S5. CF reduces UPR activation in hepatocytes, and CDN upregulates cell-surface LDLR in mice. (A,B) HuH7 cells were treated with CF (200 μM) for 24 hours. The expression of ER stress markers (pPERK, IRE1, sXBP1, GRP78, ATF4, ATF6) were measured via (A) immunoblot and (B) real-time PCR (n=5 biologically independent samples per group; data presented are mean ± s.d.). (C) 12-week-old C57BL/6J mice were treated with CDN (50 mg/kg) for 8h and fasted prior to sacrifice (n=5). Values are presented as the mean and error bars as s.d. Statistical comparisons between two groups were conducted using unpaired Student's *t*-tests (*, p<0.05; **, p<0.01; ***, p<0.001; ****, p<0.0001). Scale Bars; 50 μm. Source data are provided as a Source Data file.

Fig. S6.

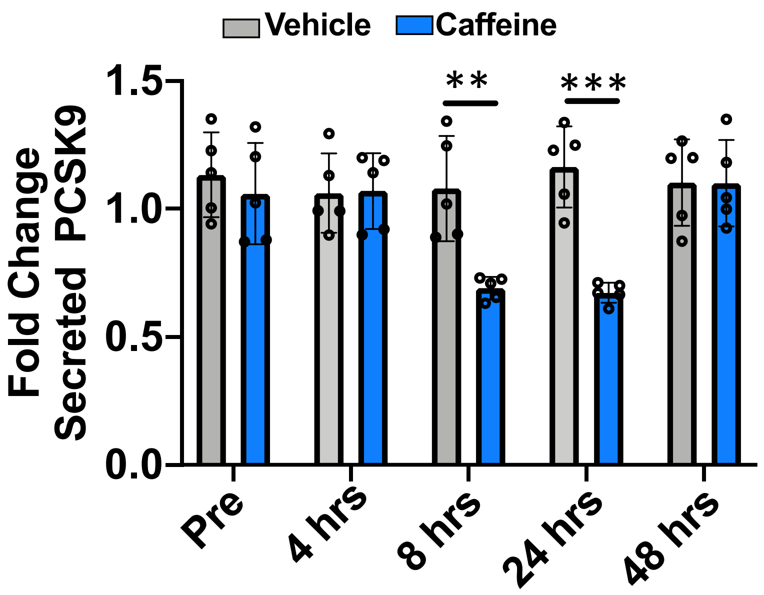

Fig. S6. Caffeine (CF) reduces plasma PCSK9 levels in mice via oral gavage. 12-week-old male C57BL/6J mice were fasted and treated with CF (20 mg/kg) of up to 48 hours prior to sacrifice (n=5). Plasma PCSK9 levels were measured at different time points using an ELISA. Values are presented as the mean and error bars as s.d. Statistical comparisons between two groups were conducted using unpaired Student's *t*-tests (*, p<0.05; **, p<0.01; ***, p<0.001). Source data are provided as a Source Data file.

Fig. S7.

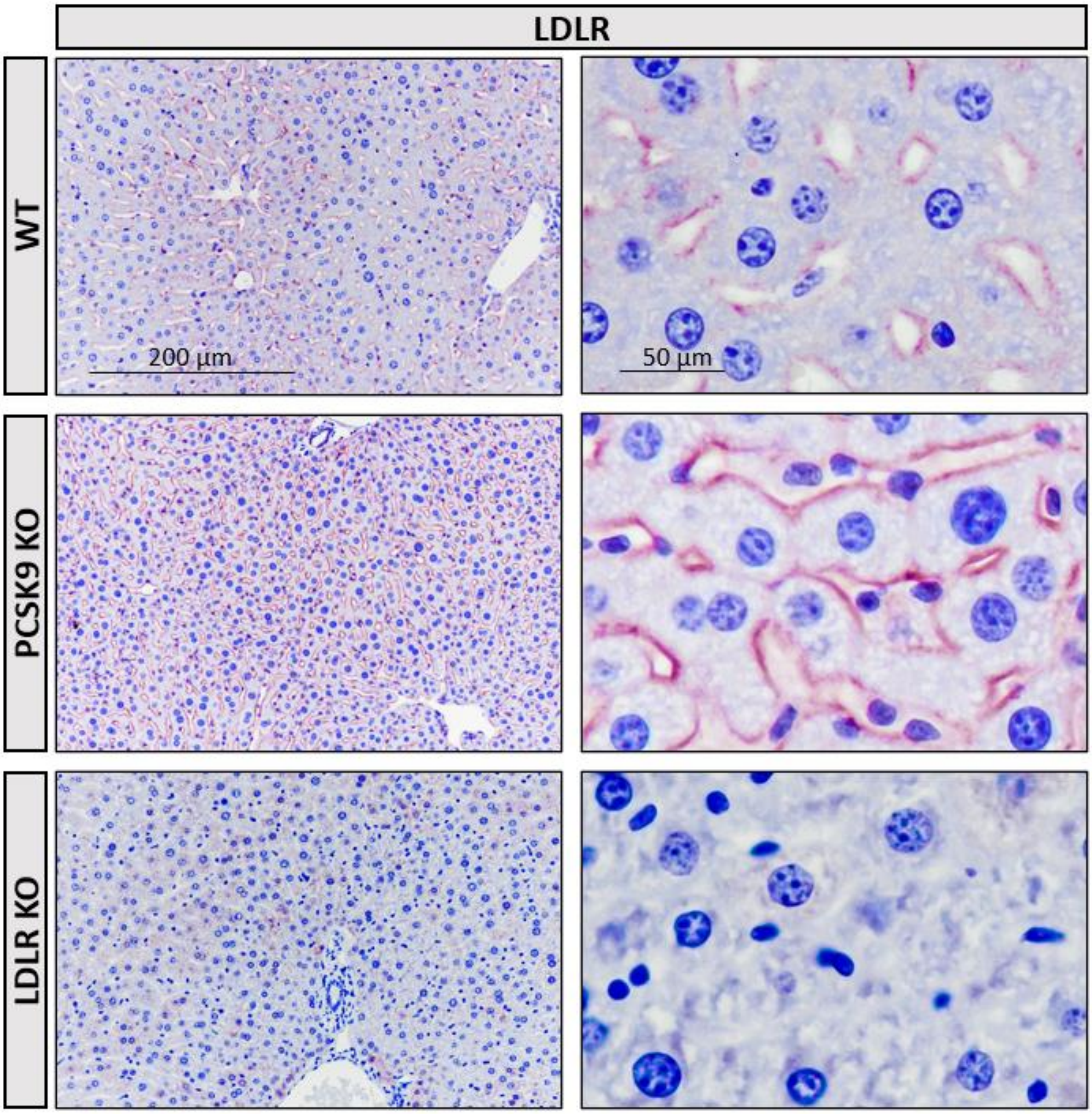

Fig. S7. 12-week-old C57BL/6J WT, LDLR knockout (KO), and PCSK9 KO mice were fasted prior to sacrifice (n=5). The livers of these mice were assessed for cell-surface expression of the LDLR via immunohistochemical staining.

Supplementary Table 1. Compounds used during the course of these studies

Compound	Effect on ER Ca^{2+} Levels	Concentrations used	Mechanism of Action
thapsigargin (TG)	decrease	100 nM	irreversible SERCA antagonist
CDN1163 (CDN)	increase	10 µM	allosteric SERCA agonist
2 APB	increase	100 µM	IP3R antagonist
ryanodine	decrease/increase	10 nM - 10 µM	RyR agonist at nM and RyR antagonist at µM
Caffeine (CF)	hypothesized increase	10 nM – 1 mM	multiple
8CC	hypothesized increase	100 nM – 1 mM	adenosine receptor antagonist
8CD	hypothesized increase	100 nM – 1 mM	adenosine receptor antagonist
PSB 603	hypothesized increase	100 nM – 1 mM	adenosine receptor antagonist
paraxanthine	hypothesized increase	100 nM – 1mM	multiple
theophyline	hypothesized increase	100 nM – 1mM	multiple
theobromine	hypothesized increase	100 nM – 1mM	multiple
Cyclopiazonic acid (CPA)	decrease	100 µM	SERCA antagonist
U18666A	N/A	10 µM	Intracellular sterol depletion

Supplementary Table 2. Chemical structures of xanthine derivatives evaluated in the study

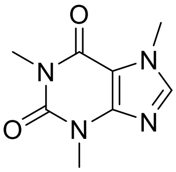

caffeine

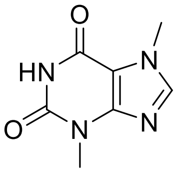

theobromine

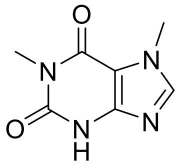

paraxanthine

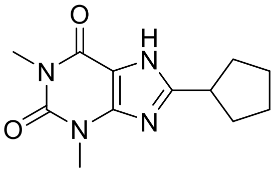

8CD
8-cyclopentyltheophylline

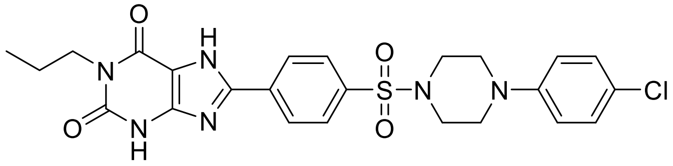

PSB603

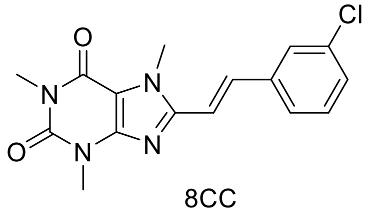

8CC
8-(3-chlorostyryl)caffeine

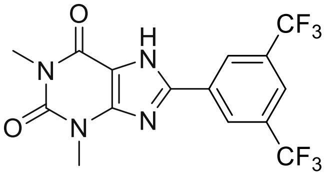

MLRA-1812

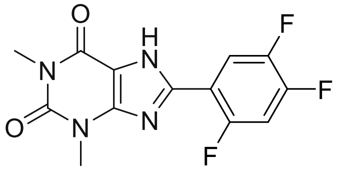

MLRA-1820

Supplementary Table 3. Antibodies used for immunoblotting and immunohistochemical staining. IB, immunoblot; IHC, immunohistochemistry; HIER, heat-induced epitope retrieval

Antibody	Catalog no.	Application	Dilution and Protocol
**β-actin**	A2228, Sigma-Aldrich	IB	1:5000
**CD36**	NB400-144, Novus Biologicals	IB	1:500
**PCSK9**	NB300-959, Novus Biologicals	IB	1:500
**SREBP-2**	557037, BD Biosciences	IB	1:500
**GFP**	NB600-308, Novus Biologicals	IB	1:1000
**Calnexin**	C5C9, Cell Signaling	IB	1:1000
**Flag**	F3165, Sigma-Aldrich	IB	1:1000
**GRP78**	610979, BD Biosciences	IB	1:1000
**GRP78**	SC-1050, Santa Cruz Biotechnology	IP	1:100
**GRP78**	Ab21685, Abcam	IHC	1:1000 HIER
**GRP94**	ADI-SPA-850, Enzo Life Sciences	IHC	1:1000, HIER
**LDLR**	AF2255, R and D Systems	IHC	1:100, HIER
**CD36**	NB400-144, Novus Biologicals	IHC	1:100, HIER

Abbreviations: IB = immunoblot; IP = immunoprecipitation; IHC = immunohistochemistry

Supplementary Table 4. Primers used for real-time PCR

Gene	Species	Forward	Reverse
**GRP78**	Mouse	GTCCTGCATCATCAGCGAAAG	GGTAGCCACATACTGAACACCA
**GRP78**	Human	CATCACGCCGTCCTATGTCG	CGTCAAAGACCGTGTTCTCG
**SREBP1**	Human	CAGGTACCGAGTTCTGGTGTGTTGGGCCA	ACTGCTAGCCGCGCTGCCGCCTCGCTAG
**SREBP1**	Mouse	ACCCTGGTGAGTGGAGGGACCATCTTGG	CTTTGCTTCAGTGCCCACCACCAGGTCTTT
**SREBP2**	Mouse	GCAGCAACGGGACCATTCT	CCCCATGACTAAGTCCTTCAACT
**SREBP2**	Human	CCTGGGAGACATCGACGAGAT	TGAATGACCGTTGCACTGAAG
**HMGR**	Mouse	CTTTCAGAAACGAACTGTAGCTCAC	CTAGTGGAAGATGAATGGACATGAT
**HMGR**	Human	TCTGGAGGATCCAAGGATTCTG	AC CAAGTGGCTGTCTCAGTGAT
**IRE1α**	Mouse	TGAAACACCCCTTCTTCTGG	CCTCCTTTTCTATTCGGTCACTT
**PCSK9**	Mouse	TGCAAAATCAAGGAGCATGGG	CAGGGAGCACATTGCATCC
**PERK**	Mouse	CCTTGGTTTCATCTAGCCTCA	ATCCAGGGAGGGGATGAT
