# Supplementary material for: Caffeine blocks SREBP2-induced hepatic PCSK9 expression to enhance LDLR-mediated cholesterol clearance
Source: Nat Commun. 2022 Feb 9;13:770. doi: 10.1038/s41467-022-28240-9 (PMC8828868; doi:10.1038/s41467-022-28240-9)
Supplement: Supplementary file 3 — Reporting Summary [file 41467_2022_28240_MOESM3_ESM.pdf]

nature portfolio

Corresponding author(s): Double-blind peer review submissions: write DBPR and your manuscript number here instead of author names. Richard C. Austin

Last updated by author(s): YYYY-MM-DD 2021-11-22

Reporting Summary

Nature Portfolio wishes to improve the reproducibility of the work that we publish. This form provides structure for consistency and transparency in reporting. For further information on Nature Portfolio policies, see our Editorial Policies and the Editorial Policy Checklist.

Statistics

For all statistical analyses, confirm that the following items are present in the figure legend, table legend, main text, or Methods section.

n/a	Confirmed	
☐	☒	The exact sample size (n) for each experimental group/condition, given as a discrete number and unit of measurement
☐	☒	A statement on whether measurements were taken from distinct samples or whether the same sample was measured repeatedly
☐	☒	The statistical test(s) used AND whether they are one- or two-sided<br>*Only common tests should be described solely by name; describe more complex techniques in the Methods section.*
☒	☐	A description of all covariates tested
☒	☐	A description of any assumptions or corrections, such as tests of normality and adjustment for multiple comparisons
☐	☒	A full description of the statistical parameters including central tendency (e.g. means) or other basic estimates (e.g. regression coefficient) AND variation (e.g. standard deviation) or associated estimates of uncertainty (e.g. confidence intervals)
☐	☒	For null hypothesis testing, the test statistic (e.g. F, t, r) with confidence intervals, effect sizes, degrees of freedom and P value noted<br>*Give P values as exact values whenever suitable.*
☒	☐	For Bayesian analysis, information on the choice of priors and Markov chain Monte Carlo settings
☒	☐	For hierarchical and complex designs, identification of the appropriate level for tests and full reporting of outcomes
☒	☐	Estimates of effect sizes (e.g. Cohen's d, Pearson's r), indicating how they were calculated

Our web collection on statistics for biologists contains articles on many of the points above.

Software and code

Policy information about availability of computer code

Data collection	No software was used for data collection
Data analysis	Excel (v.16 for Office 365); ImageJ (v.1.8.0_172)

For manuscripts utilizing custom algorithms or software that are central to the research but not yet described in published literature, software must be made available to editors and reviewers. We strongly encourage code deposition in a community repository (e.g. GitHub). See the Nature Portfolio guidelines for submitting code & software for further information.

Data

Policy information about availability of data

All manuscripts must include a data availability statement. This statement should provide the following information, where applicable:

- Accession codes, unique identifiers, or web links for publicly available datasets
- A description of any restrictions on data availability
- For clinical datasets or third party data, please ensure that the statement adheres to our policy

No large databases were used in this manuscript; there are no restrictions on the availability of the data in this manuscript

Field-specific reporting

Please select the one below that is the best fit for your research. If you are not sure, read the appropriate sections before making your selection.

[x] Life sciences [ ] Behavioural & social sciences [ ] Ecological, evolutionary & environmental sciences

For a reference copy of the document with all sections, see nature.com/documents/nr-reporting-summary-flat.pdf

Life sciences study design

All studies must disclose on these points even when the disclosure is negative.

Sample size	No sample size calculation was conducted ; Sample size was determined based on past experiences with experimental models and treatments
Data exclusions	No data exclusions were implemented in this manuscript
Replication	All experiments were repeated at least three times; the data shown in the manuscript are representative of at least three successful experiments
Randomization	All cells and mice used in the study were randomly allocated to treatment groups<br>Study conducted in healthy volunteers was based on a single arm protocol and did not require randomization
Blinding	Scientists were not blinded for in vivo studies involving mice<br>Scientists were not blinded for clinical studies due to single arm protocol

Reporting for specific materials, systems and methods

We require information from authors about some types of materials, experimental systems and methods used in many studies. Here, indicate whether each material, system or method listed is relevant to your study. If you are not sure if a list item applies to your research, read the appropriate section before selecting a response.

Materials & experimental systems

n/a	Involved in the study	
[ ]	[x]	Antibodies
[ ]	[x]	Eukaryotic cell lines
[x]	[ ]	Palaeontology and archaeology
[ ]	[x]	Animals and other organisms
[ ]	[x]	Human research participants
[x]	[ ]	Clinical data
[x]	[ ]	Dual use research of concern

Methods

n/a	Involved in the study	
[x]	[ ]	ChIP-seq
[x]	[ ]	Flow cytometry
[x]	[ ]	MRI-based neuroimaging

Antibodies

Antibodies used

1. anti-PCSK9: Novus Biologicals, NB300-959
2. anti-SREBP2: Abcam, ab30682
3. anti-GFP: Novus Biologicals, NB600-308
4. anti-Beta Actin: Sigma Aldrich, A222
5. anti-Calnexin: Cell Signaling Technology, C5C9
6. anti-GRP78: BD Biosciences, 610979
7. anti-LDLR: R and D Systems, AF2255
8. anti-IRE1: Cell Signaling Technology, 4668
9. anti-GRP94: Enzo Life Sciences, ADI-SPA-850
10. anti-CHOP: Santa Cruz Biotechnology, SC-575
11. anti-GAPDH: Cell Signaling Technology, 14C10
12. anti-CD36: Novus Biologicals, NB400-144
13. anti-GRP78: Santa Cruz Biotechnology, SC-1050
14. anti-GRP78: Abcam, Ab21685

Validation

1. Used for WB in: Lebeau P, Platko K, Al-Hashimi AA et al. Loss-of-function PCSK9 mutants evade the unfolded protein response sensor, GRP78, and fail to induce endoplasmic reticulum stress when retained. J. Biol. Chem. Mar 28 2018
2. Used in WB: Wei S, Liu L, Chen Z, Yin W, Liu Y, Ouyang Q, Zeng F, Nie Y, Chen T. Artesunate inhibits the mevalonate pathway and promotes glioma cell senescence. J Cell Mol Med. 2020 Jan;24(1):276-284. doi: 10.1111/jcmm.14717.
3. Used in WB: Yu D, Febbo IG, Maroteaux MJ, Wang H, Song Y, Han X, Sun C, Meyer EE, Rowe S, Chen Y, Canavier CC, Schrader LA. The Transcription Factor Shox2 Shapes Neuron Firing Properties and Suppresses Seizures by Regulation of Key Ion Channels in Thalamocortical Neurons. Cereb Cortex. 2021 Jun 10;31(7):3194-3212. doi: 10.1093/cercor/bhaa414.
4. Used in WB: Kanakkanthara, A., Wilmes, A., Obrate, A., Escuin, D., Chan, A., Gjyrezi, A., ... Miller, J. H. (2011). Peloruside- and

Laulimalide-Resistant Human Ovarian Carcinoma Cells Have I-Tubulin Mutations and Altered Expression of II- and III-Tubulin Isotypes. Molecular Cancer Therapeutics, 10(8), 1419–1429. doi: 10.1158/1535-7163.mct-10-1057
5. Used in WB: He C, Hua W, Liu J, Fan L, Wang H, Sun G. Exosomes derived from endoplasmic reticulum-stressed liver cancer cells enhance the expression of cytokines in macrophages via the STAT3 signaling pathway. Oncol Lett. 2020 Jul;20(1):589-600. doi: 10.3892/ol.2020.11609.
6. Used in WB: Han JM, Kim Y, Lee JS. Localization of phospholipase D1 to caveolin-enriched membrane via palmitoylation: implications for epidermal growth factor signaling. Mol Biol Cell. 2002; 13(11):3976-3988.
7. Used in WB: Lebeau, P., Platko, K., Al-Hashimi, A., Byun, J. H., Lhotak, S., Holzapfel, N., … Austin, R. C. (2018). Loss-of-function PCSK9 Mutants Evade the Unfolded Protein Response Sensor, GRP78, and Fail to Induce Endoplasmic Reticulum Stress when Retained. Atherosclerosis Supplements, 32, 139. doi: 10.1016/j.atherosclerosissup.2018.04.426
8. Used in WB: Shin, G.-C., Moon, S. U., Kang, H. S., Choi, H.-S., Han, H. D., & Kim, K.-H. (2019). PRKCSH contributes to tumorigenesis by selective boosting of IRE1 signaling pathway. Nature Communications, 10(1). doi: 10.1038/s41467-019-11019-w
9. Used in WB: Fu, Y.-L., Zhang, B., & Mu, T.-W. (2019). LMAN1 (ERGIC-53) promotes trafficking of neuroreceptors. Biochemical and Biophysical Research Communications, 511(2), 356–362. doi: 10.1016/j.bbrc.2019.02.053
10. Used in WB: Imbernon, M. et al. 2016. Hepatology (Baltimore, Md.). 64: 1086-104.
11. Used in WB: Cai H, Ren L, Wang Y, Zhang Y. Beta-Elemene Reduces the Malignancy of Non-Small Cell Lung Cancer by Enhancing C3orf21 Expression. Front Oncol. 2021 May 7;11:571476. doi: 10.3389/fonc.2021.571476.
12. Used in WB: Huang, L;Chambliss, KL;Gao, X;Yuhanna, IS;Behling-Kelly, E;Bergaya, S;Ahmed, M;Michaely, P;Luby-Phelps, K;Darehshouri, A;Xu, L;Fisher, EA;Ge, WP;Mineo, C;Shaul, PW; SR-B1 drives endothelial cell LDL transcytosis via DOCK4 to promote
13. Used in 83 publications based on manufacturer website; 14. Used in 459 publications based on manufacturer website

Eukaryotic cell lines

Policy information about cell lines

Cell line source(s)	1. HEPG2 (ATCC; HB-8065)<br>2. HuH7 (kind gift from collaborator)
Authentication	None of the cell lines were authenticated, but were used in several cited reports.
Mycoplasma contamination	Both cell lines are negative for Mycoplasma contamination.
Commonly misidentified lines (See ICLAC register)	No commonly misidentified cell lines were used in the study

Animals and other organisms

Policy information about studies involving animals; ARRIVE guidelines recommended for reporting animal research

Laboratory animals	Male C57BL/6J mice that were either 12 or 18 weeks of age were used in this study. Male Pcsk9-/- and Ldlr -/- on the C57BL/6 background were also used for control experiments.
Wild animals	No wild animals were used in this study
Field-collected samples	The study did not involve field-collected samples
Ethics oversight	All animal studies were approved by the McMaster University ethics board

Note that full information on the approval of the study protocol must also be provided in the manuscript.

Human research participants

Policy information about studies involving human research participants

Population characteristics	No information on co-variates was collected. The study had no restriction regarding inclusion/exclusion criteria.
Recruitment	Participants were recruited using flyers posted near the laboratory ; random selection of patients was used to minimize any potential biases
Ethics oversight	The human study included in this manuscript was approved by the Hamilton Integrated Research Ethics Board (HIREB; project number 5805)

Note that full information on the approval of the study protocol must also be provided in the manuscript.
